# Effects of natural polyphenol-rich pomegranate juice on the acute and delayed response of Homocysteine and steroidal hormones following weightlifting exercises: a double-blind, placebo-controlled trial

**DOI:** 10.1186/s12970-020-00345-w

**Published:** 2020-03-06

**Authors:** Achraf Ammar, MounaTurki, Khaled Trabelsi, Nicola Luigi Bragazzi, Omar Boukhris, Mohamed Bouaziz, Fatma Ayadi, Kais El Abed, Tarak Driss, Nizar Souissi, Hamdi Chtourou, Stephen J. Bailey, Anita Hoekelmann

**Affiliations:** 1 Institute of Sport Sciences, Otto-von-Guericke University, 39104 Magdeburg, Germany; 2 Unit of Research Molecular Bases of Human Diseases, 12ES17, Faculty of Medicine of Sfax, University of Sfax, 3000 Sfax, Tunisia; 3 Laboratory of Biochemistry, CHU Habib Bourguiba, Sfax University, 3000 Sfax, Tunisia; 4 High Institute of Sport and Physical Education, University of Sfax, 3000 Sfax, Tunisia; 5 Department of Health Sciences (DISSAL), Postgraduate School of Public Health, University of Genoa, 16132 Genoa, Italy; 6 High Institute of Biotechnology, Sfax University, 3000 Sfax, Tunisia; 7 Interdisciplinary Laboratory in Neurosciences, Physiology and Psychology: Physical Activity, Health and Learning (LINP2-2APS), UFR STAPS, UPL, Paris Nanterre University, Nanterre, France; 8 Activité Physique, Sport et Santé, UR18JS01, Observatoire National du Sport, 1003 Tunis, Tunisia; 9 School of Sport, Exercise and Health Sciences, Loughborough University, Loughborough, LE11 3TU UK

**Keywords:** Supplementation, Testosterone, Cortisol, Cardio-vascular health, Antioxidant, Strength

## Abstract

**Background:**

Maximal strength-speed exercise is a powerful stimulus to acutely increase concentrations of circulating steroid hormones and homocysteine [Hcy]. There is some evidence that antioxidant beverages rich in polyphenols can attenuate [Hcy] levels and modulate endocrine responses in favor of an anabolic environment. Polyphenols-rich pomegranate (POM) have been reported to possess one of the highest antioxidant capacities compared to other purported nutraceuticals and other food stuffs. Studies focused on proving the beneficial effect of POM consumption during maximal strength exercises have only measured physical performance, muscle damage, oxidative stress and inflammatory responses, while POM effects on [Hcy] and hormonal adaptations are lacking. The aim of the present study was to investigate the effect of consuming natural polyphenol-rich pomegranate juice (POMj) on the acute and delayed [Hcy] and steroidal hormonal responses to a weightlifting exercises session.

**Methods:**

Nine elite weightlifters (21.0 ± 1 years) performed two Olympic-weightlifting sessions after ingesting either the placebo (PLA) or POMj supplements. Venous blood samples were collected at rest and 3 min and 48 h after each session.

**Results:**

Compared to baseline values, circulating cortisol [C] decreased (*p* < 0.01) and testosterone/cortisol [T/C] ratio increased immediately following the training session in both PLA and POMj conditions (*p* = 0.003 for PLA and *p* = 0.02 for POM). During the 48 h recovery period, all tested parameters were shown to recover to baseline values in both conditions with significant increases in [C] and decreases in [T/C] (*p* < 0.01 for PLA and *p* < 0.05 for POMj) from 3 min to 48 h post-exercises. Compared to PLA, a lower level of plasma testosterone [T] was registered 3 min post exercise using POMj supplementation (*p* = 0.012) and a significant decrease (*p* = 0.04, %change = − 14%) in plasma [Hcy] was registered during the 48 h recovery period only using POMj. A moderate correlation was observed between [Hcy] and [T] responses (*p* = 0.002, *r* = − 0.50).

**Conclusion:**

In conclusion, supplementation with POMj has the potential to attenuate the acute plasma [T] response, but did not effect 48 h recovery kinetics of [Hcy] following weightlifting exercise. Further studies investigating androgen levels in both plasma and muscular tissue are needed to resolve the functional consequences of the observed acute POMj effect on plasma [T].

**Trial registration:**

Clinical Trials.gov, ID: NCT02697903. Registered 03 March 2016**.**

## Introduction

Pomegranate (POM) or *Punica granatum* is an ancient fruit originating from the Middle East [1]. Pomegranate is composed of peel (mesocarp and endocarp; ≈ 50% of the total fruit weight) and the edible parts (arils, seeds; ≈ 50% of the total fruit weight) with the highest chelating and scavenging capacity (37.22%), and the highest oxygen radical absorbance capacity (55,520 μmol TE/100 g) found in peels [1, 2]. Compared to other purported nutrients (e.g., green tea, red wine, orange, blueberry and cranberry juices), and other food stuffs (e.g., turmeric, ragi, amla, amaranth, rajmah, sesame, wheat and flaxseed), polyphenol-rich POM supplements have been reported to possess one of the highest antioxidant capacities [3, 4] with a trolox equivalent antioxidant capacity three-time higher than green tea and red wine [4]. This potent antioxidant activity of POM has been attributed to the high bioavailability of its polyphenols and other biologically active compounds (e.g., flavonols, flavanoids, gallicacid, ellagic acid, quercetin, ellagitannins, and nitrate) compared to the aforementioned polyphenol-rich foods [4, 5]. In individuals exhibiting physiological stress such as cardiovascular disease (CVD) [6], oxidative stress [7], cellular inflammation or joint or muscle damage [8–11], the consumption of POM supplementation appears to exhibit a high capacity to neutralize free radicals and to promote several beneficial health effects [5, 12]. In particular, polyphenol-rich POM supplementation appears to lower CVD risk factors by attenuating low density lipoprotein oxidation and carotid artery thickness [6] and enhancing myocardial blood flow [13] and antioxidant status [6]. Additionally, it has been shown that POM supplementation is an effective inhibitor of some cellular inflammation transcripts such as tumor necrosis factor α, nuclear factor -κB and cyclooxygenase-2 [14, 15]. Similarly, POM supplementation has been shown to regulate expression of androgen-synthesizing genes in human prostate cancer cells [16] and to modulate hormonal profile by reducing cortisol [C] urinary output and fasting plasma insulin in volunteers at high CVD risk [17] and increasing testosterone [T] levels in testis torsion Wistar rats [18] and healthy humans [19].

Since POM supplementation has the potential to enhance physiological responses of people manifesting symptoms of physiological stress [5–9], and as intensive physical exercise is a potent and multifaceted physiological stressor [20–23], previous reports have suggested POM supplementation as an effective ergogenic and recovery aid for athletic populations [24, 25]. The effectiveness of POM supplementation on exercise performance and post-exercise recovery has been shown by numerous studies [24–33]. Indeed, POM supplementation appears to hold potential as a nutritional aid to enhance performance during and alleviate muscle fatigue and soreness following endurance [27–29] and strength [10, 24, 25, 31] exercise. POM also appears to enhance post exercise recovery of skeletal muscle function during different type of exercises [11] by attenuating muscle damage following weightlifting exercise [10], promoting antioxidant defenses following exhaustive strength exercises [26] and aerobic based-exercises [30, 32, 33], mitigating inflammation during exhaustive running exercise [32] and enhancing cardiovascular function during strength [10, 30] and treadmill running exercise [29]. These ergogenic and recuperative effects of POM supplementation have been purported to be linked to the potent free-radicals-scavenging effect of POM polyphenols [34] and to its potential to promote vasodilation, which improve nutrient delivery to and promote the efflux of noxious metabolic by-products from skeletal muscle [29, 35].

Strength exercise is one of the best training modalities to enhance maximal force and power output [36]. This exercise leads to an elevation of protein synthesis, activation of satellite cells, muscle cell signaling pathways and hormone responses [37]. Similarly, multiple physiological strain responses such as acute and delayed increases in muscle damage, oxidative stress and inflammation [20–23] have been also reported during strength exercise and particularly during Olympic weightlifting exercise. Therefore, weightlifting exercise, which is well known to elicit one of the highest peak power outputs and to requires high metabolic cost [38–41], was suggested to be the best exercise modality to test the efficacy of POM as an ergogenic and recovery aid supplementation during intensive exercise [10]. Maximal strength-speed exercise is also a powerful stimulus to acutely increase concentrations of circulating [T], [C] and homocysteine [Hcy] [42–44]. There is some evidence that antioxidant beverages rich in polyphenols can attenuate [Hcy] levels in Alzheimer disease patients [45] and modulate endocrine responses in favor of an anabolic environment in healthy [19] and diseased [46] individuals. Studies focused on proving the beneficial effect of POM consumption during weightlifting exercises have only measured Olympic performance, muscle damage, oxidative stress and inflammatory responses [10, 26], while POM effects on [Hcy] and hormonal adaptations are lacking. Moreover, information pertaining to [T], [C] and their respective ratio [T/C] may be used to detect the imbalance between anabolic and catabolic metabolism and its subsequent effect on strength and muscle adaptations associated with resistance training [47].

To the best of the authors’ knowledge, no previous studies have examined the influence of POM supplementation on [Hcy] and hormonal responses during intensive strength exercise. Therefore, the aim of the present study was to investigate the influence of natural polyphenol-rich pomegranate juice (POMj) supplementation on acute and delayed [Hcy] and steroidal hormonal responses following an intensive weightlifting training session. We hypothesized that the repeated consumption of POMj during the 48 h proceeding the training session mixed with an acute consumption 1 h prior exercises would reduce the acute and delayed responses of [Hcy] and modulate the responses of steroidal hormones in favor of an anabolic environment.

## Materials and methods

### Participants selection: inclusion and exclusion criteria

The sample size was calculated a priori, using procedures suggested by Beck [48] and the software G*Power [49]. Values for α were set at 0.05 and for power at 0.90. Based on the results of Beyer et al. [50], effect sizes were estimated to be 0.53 (medium effect). To reach the desired power, data from at least eight participants were deemed to be sufficient to minimize the risk of incurring a type 2 statistical error.

Nine elite male weightlifters [21 ± 1 years, 80 ± 10 kg, 1.75 ± 0.08 m (mean ± SD)] volunteered to participate in this study. The participants were recruited on the basis of: (i) they trained at least five sessions/week, (ii) they had at least 3 years of experience in Olympic weightlifting, (iii) they did not have any injury, (iv) they did not use any antioxidant or anti-inflammatory drugs, and (v) they did not consume foods rich in antioxidants or polyphenols (e.g., coffee, blueberries, grapes, tea, cherries, red wine, dark chocolate and curcuma [11]) during the experimental period and for at least 1 month prior to the commencement of the study [10, 26]. Each participant provided their written informed consent to take part in the experiment after receiving a thorough explanation of the possible risks and discomforts associated with the experimental procedures.

The study was conducted according to the Declaration of Helsinki and the study’s protocols and procedures were fully approved (identification code: 8/16) by the local ethics committee of “Habib Bourguiba hospital, Sfax, Tunisia” before the commencement of the assessments. Additionally, all ongoing and related trials for this intervention were registered with Clinical Trials.gov (identification code: NCT02697903, Registered 03 March 2016 - Retrospectively registered, https://clinicaltrials.gov/ct2/show/NCT02697903).

### Experimental design

A double blind, placebo-controlled design was adopted for this study. Neither staff nor participants were informed about the names of the two drinks, and blinding was strictly maintained by emphasizing to both staff and participants that both drinks were health promoting and advocated as potentially performance enhancing by certain sports medicine experts.

One week before the beginning of the experimental period, the heaviest weight lifted in a single repetition (1-Repetition Maximum (1-RM)) was assessed for each participant in each Olympic movement. 1-RM was determined in three trials interspersed by 5 min recovery after an ascending warm-up from 40 to 80% of the athlete’s estimated maximum [10, 20]. Thereafter, participants performed, as part of their habitual training-program to avoid any repeated bout effect, two training sessions from 08:00 to 09:45 following the consumption of PLA and POMj with a 48 h washout between conditions [10, 26]. Each training session comprised three Olympic Weightlifting exercises (snatch, clean and jerk, and squat) with 5 sets for each exercise and a total session duration of 1 h 46 min. Specifically, participants completed 2 sets of 3 repetitions at 85% of 1-RM and 3 sets of 2 repetitions at 90% of 1-RM) [20–23]. A passive recovery period of 5 and 8 min was administered between sets and the different Olympic-Weightlifting exercises [20–23].

Participants consumed 250 mL of the PLA or POMj supplementations three times daily over the 48 h that proceeded the two training sessions with 8 h intervals between each 250 mL serving of the supplement. Moreover, participants consumed an additional opaque and unmarked 500 mL can of PLA or POMj 60 min before commencing the training sessions [10, 26] to facilitate beneficial physiological effects [11]. The two drinks were similar in volume, texture, and appearance. Participants were instructed to drink the fluid quickly (within 1 ± 0.5 min) 60 min before their test session and not to discuss or compare tastes or to make any assumptions about what they had ingested. The interval of 60 min was chosen as optimal for a complete polyphenol absorption and thus favoring the attainment of peak polyphenol concentrations [11]. Before and after (at 3 min and at 48 h post-training session) each training session, blood samples were collected. Before test sessions, participants underwent an overnight fast and were only permitted to drink one glass of water (15–20cL) to avoid the potential confounding influence of postprandial thermogenesis [20–23].

Given that randomly assigning the supplements would have resulted in some participants consuming the POMj supplement before the PLA supplement, and since the beneficial effects of POMj could persist for up to 3 weeks after consumption [51], we elected to avoid any potential confounding effect of POMj supplementation altering blood responses during the PLA condition by ensuring all participants completed the PLA condition first (non-randomized order) [10, 11, 26]. As the participants were well trained and familiar with the exercises, and as the protocol was completed by all participants during the regular training program, in the same hours of their regular training session (to avoid any time of day effect [22, 23]) and in the middle of the precompetitive period, the authors assume that an order effect is less likely to have occurred [26].

### Pomegranate juice and placebo supplementations

Beverages were prepared by an agri-food engineer. The natural POMj was prepared from a fresh pomegranate fruit 48 h before the beginning of the experimentation and was frozen and stored at − 4 °C. No additional chemical products were added to the natural POMj. PLA juice consisted of a pomegranate-flavored drink containing mineral water, natural identical flavor (pomegranate), stabilizers (Arabic-gum) with caloric sweeteners (i.e., added sugar) added to match the POMj energy content and avoid any confounding effect of different caloric content of the beverages [52]. To the authors’ knowledge, there is no evidence to suggest that artificial sweeteners or stabilizers (included in PLA) could modulate hormonal profile or [Hcy] compared to natural sugar (included in POMj) and thereby impact the present results. Indeed, both natural sugars and artificial sweeteners are rich in calories have been shown to raise blood sugar levels [53].

Concerning the POMj processing, a previous study showed that the polyphenol content of POMj may vary between 900 and 2300 mg/L [54, 55] when varying the peels and arils proportions with an increasing proportion of peels (containing higher polyphenols and antioxidant contents) resulting in increased polyphenol content [54]. Therefore, to insure a high proportion of peels (≥15%) and thereby a high polyphenol content (> 1.69 g/L as suggested by Ammar et al. [11]), the agri-food engineer manually prepared the POMj to be administered from the whole fresh POM by tightly squeezing each half-POM (squeezing the edible portion and a part of the peels portion) using a plain juicer.

One can of POMj (i.e., 500 mL) contained 2.56 g of total polyphenols, 1.08 g of orthodiphenols, 292.6 mg of flavonoids and 46.75 mg of flavonols, 64 g of total carbohydrates (i.e., 56 g of sugar), and 1046 kJ of energy. The PLA drink (i.e., 500 mL) did not contain antioxidants, vitamins or polyphenols, but comprised 60 g total carbohydrates (i.e., 54 g of added sugar), and 983 kJ of energy (Table 1).

**Table 1:** Nutrition Facts of Pomegranate and Placebo Juices

Variables	Contents / 500 ml	
	POMj	PLA
Total polyphenols (g)	2,56	0
Orthodiphenols (g)	1,08	0
Flavonoids (mg)	292,6	0
Flavonols (mg)	46,75	0
Calories	250	235
Calories from Fat	0	0
Total Fat (g)	0	0
Saturated Fat (g)	0	0
Trans Fat (g)	0	0
Cholestreol (mg)	0	0
Total Carbohydrate (g)	64	60
Dietery Fiber (g)	0	0
Sugars (g)	56	54
Protein (g)	< 1	0
Vitamin A (%)	0	0
Vitamin C (%)	0	0
Iron (%)	0	0

The phenolic extracts were obtained following the procedure of Chtourou et al. [56] with some modifications. Firstly, the POMj sample (4 g) was added to 2 mL of n-hexane and 4 mL of a methanol/water (60:40, v/v) mixture in a 20 mL centrifuge tube. After vigorous mixing, the mixture was centrifuged for 3 min at 1490×g. The hydroalcoholic phase was collected, and the hexane phase was re-extracted twice with 4 mL of the methanol/water (60:40, v/v) solution each time. Finally, the hydro-alcoholic fractions were combined, washed with 4 mL of n-hexane to remove the residual POMj, then concentrated and dried by evaporative centrifuge in vacuum at 35 °C.

The determination of the total phenolic compounds was performed by means of the Folin-Ciocalteau reagent using the method described by Gargouri et al. [57]. The total phenolic content was expressed as milligrams of gallic acid (GA) equivalent per kilogram of POMj (y = 0.011 x, R^2^ = 0.990). The absorbance was measured at λ = 765 nm, using a spectrophotometer (Shimadzu UV-1800 PC, Japan). The concentration of o-diphenolic compounds in the methanolic extract was determined by the method of Dridi-Gargouri et al. [58]. The total o-diphenolic content was expressed as milligrams of GA equivalent per kilogram of POM (y = 1.144 x, R^2^ = 0.999). The absorbance was measured at λ = 370 nm, using the same spectrophotometer.

Total flavonoids were measured by a colorimetric assay developed by Gargouri et al. [57]. Specifically, a 1 mL aliquot of appropriately diluted sample or standard solutions of catechin (20, 40, 60, 80 and 100 mgL^− 1^) was added to a 10 mL volumetric flask containing 4 mL double-distillate H_2_O. At zero-time, 0.30 mL 5% NaNO_2_ was added to the flask. After 5 min, 0.30 mL 10% AlCl_3_ was added. At 6 min, 2 mL (1 molL^− 1^) NaOH was added to the mixture. The reaction flask was then immediately diluted by the addition of 2.4 mL of double-distilled H_2_O and thoroughly mixed. The absorbance of the mixture was determined at 510 nm after correction for a water blank. The total flavonoids in the POMj was expressed as mg^.^100 g^− 1^ fresh weight catechin equivalents. Samples were analyzed in triplicate.

### Dietary records

To assess the adequacy of nutrient intake, a consecutive dietary record over 7 days was completed. All participants received a detailed verbal explanation and written instructions on how to record their diet over the study period. Participants were asked to continue with their usual dietary habits during the period of dietary recording and to be as accurate as possible in recording the amounts and types of food and fluid consumed. A list of common household measures (e.g., tablespoons, cups), and specific information about the quantity in each measurement (grams, etc.) were given to each participant. Each individual’s dietary composition was estimated using the Bilnut 4 software package (SCDA Nutrisoft, Cerelles, France) and the food composition tables published by the Tunisian National Institute of Statistics in 1978. Estimated nutrient intakes were referred to reference dietary intakes for physically active people and the daily nutriment data showed that total calorie, macronutrient, and micronutrient intakes were within expected ranges for healthy Tunisian adults.

### Blood sampling and analysis

Blood samples (7 mL) were collected from a forearm vein before, 3 min and 48 h following each training session in the PLA and POMj conditions. Samples were placed in an ice bath and immediately centrifuged at 2500 rpm and 4 °C for 10 min. Aliquots of the resulting plasma were stored at − 80 °C until analysis. To eliminate inter-assay-variance, all samples were analyzed in the same assay run. All assays were performed in-duplicate in the same laboratory with simultaneous use of a control serum from Randox. Levels of plasma [Hcy], [T] and [C] were determined using Architect Ci 4100 d’ABOTT. The intra-assay and inter-assay coefficients of variation were < 4% for all assays performed. The ratio testosterone/cortisol [T/C] was derived from the respective concentrations. Additionally, to account for any change in plasma volume shifts post exercise, hematological parameters [i.e., Red Blood Cells (RBC), Hemoglobin (HB), Hematocrit (HCT)] were assessed within 3 h in a multichannel automated blood cell analyzer (Beckman Coulter Gen system-2, Coulter T540, California, United State).

### Statistical analysis

All statistical analyses were performed using STATISTICA 10.0 Software. Normality of the data distribution was confirmed using the Shapiro-Wilks-W-test. To analyze the effect of POMj supplementation on the [Hcy] and steroidal responses during training sessions (pre-post values), a two-way [supplement (PLA and POMj) × time (pre and 3 min post training session)] ANOVA with repeated measures was employed. To analyze the effect of POMj supplementation on the recovery kinetics of the selected parameters, a one-way ANOVA was utilized. When significant main effects were observed, Tukey’s honest-significance-difference (HSD) post-hoc tests were conducted. The effect size (ES) for each studied interaction in ANOVA analysis was calculated using the eta squared (η^2^) by using between-groups sum of squares and the total sums of squares for all ES. The magnitude of η2 was interpreted following the Cohen’s guidelines as follows: small: 0.01, medium = 0.06 and large: 0.14. For each studied main effect in ANOVA analysis, ES was calculated as partial eta-squared (η_p_^2^) and interpreted for the ANOVA analysis to interpret determine the magnitude of the change score and was assessed using the following criteria: < 0.2 = trivial, 0.2–0.6 = small, 0.6–1.2 = moderate, 1.2–2.0 = large, 2.0–4.0 = very large, and > 4.0 = extremely large [59]. To assess the correlation between the [Hcy] and the steroidal hormones measures, Bland-Altman correlation (1995) was performed using MedCalc software (v9.0.1.1; MedCalc Software, Mariakerke, Belgium). The magnitude of correlation was interpreted as: low: < 0.3; moderate: < 0.5; high: < 0.7; and, very high: > 0.7. Statistical significance was set at *p* < 0.05 and data are presented as mean ± SD unless otherwise stated.

## Results

### Hematological parameters cell count

Compared with the baseline values, there was no significant change in the hematological parameters (i.e., RBC, HB and HCT) in response to the weightlifting exercises in both PLA and POMj conditions (Table 2). Additionally, there was no significant difference between supplementation conditions at any of the time-points. Therefore, any observed changes in the blood parameters assessed (i.e., Hcy, T and C) in response to the weightlifting exercises or POMj supplementation cannot be attributed to changes in hemoconcentration [60].

**Table 2:** Hematological parameters before, immediately (3 min) and 48 h after the PLA and POMj resistance training sessions

Parameters	PLA			POMj		
	Basal	3 min	48 h	Basal	3 min	48 h
***RBC (10***^***6***^***/ μl)***	5.60 ± 0.21	5.68 ± 0.18	5.43 ± 0.14	5.43 ± 0.14	5.51 ± 0.20	5.55 ± 0.10
***Hemoglobin (g/dl)***	15.68 ± 1.33	16.30 ± 1.95	15.96 ± 1.36	15.96 ± 1.36	16.06 ± 1.49	16.28 ± 1.25
***Hematocrit (%)***	48.53 ± 2.02	48.94 ± 2.57	47.71 ± 2.10	47.71 ± 2.10	48.19 ± 2.36	48.47 ± 1.82

### Effect of POMj on the acute responses of plasma Hcy and steroidal hormones immediately following the weightlifting training session

**Fig. 1:**
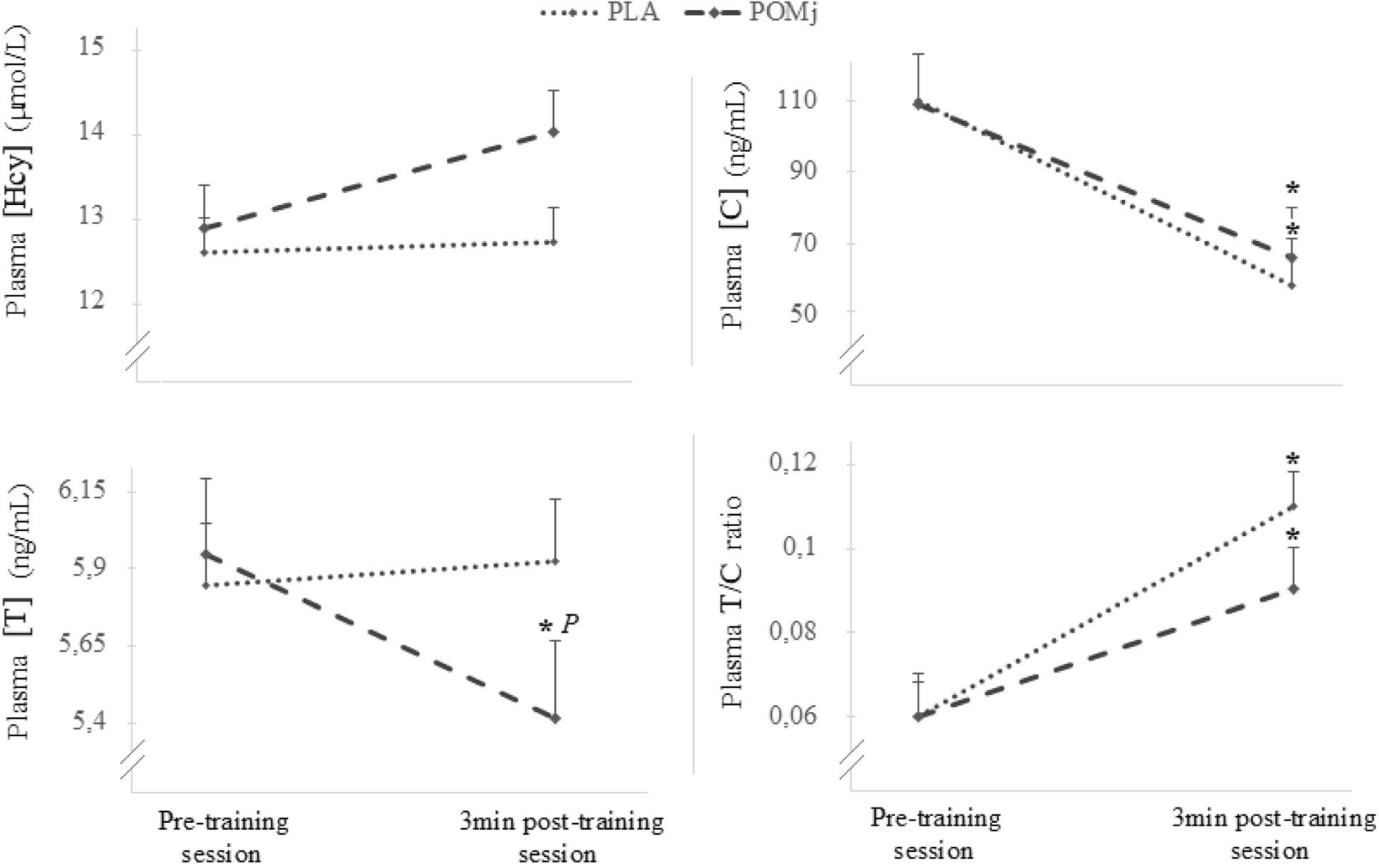
Acute homocysteine, steroidal hormones (i.e., cortisol (C) and testosterone (T)) and T/C ratio responses to a weightlifting training session following placebo (PLA) and pomegranate (POMj) supplementation. *: Significant differences between pre- and post-training session. *P*: Significant difference between PLA and POMj condition

Mean and SD values for the steroidal hormones (i.e.*,* [T] and [C]), the T/C ratio and the [Hcy] before and after the weightlifting sessions (i.e.*,* using PLA and POMj conditions) are presented in Fig. 1. Statistical analysis showed a significant interaction (training session × supplementation condition) for [T] (F_(1,8)_ = 7.48, *p* = 0.026 η^2^ = 0.05) with a significant decrease pre-post training session only registered during the POMj condition (*p* = 0.009, % change = − 9.8 ± 3.9%), resulting in lower post-session values of [T] during the POMj condition compared to the PLA condition (*p* = 0.012).

Significant main effect of the training session was observed for both [C] (F_(1,8)_ = 99.78, *p* = 0.000 ηp^2^ = 0.93) and [T/C] (F_(1,8)_ = 63.64, *p* = 0.000 ηp^2^ = 0.89) parameters with [C] level significantly decreased from pre- to post- training session in both PLA (*p* = 0.003, % change = − 42.2 ± 28.3%) and POMj (*p* = 0.007, % change = − 40.1 ± 19.6%) conditions; while [T/C] level significantly increase immediately after the training session (*p* = 0.003, % change = 122.1 ± 106.5% for PLA and (*p* = 0.02, % change = 74.6 ± 56.6%) for POMj. However, no significant difference between both conditions was registered for [C] and [T/C] ratio 3 min post training session (*p* > 0.05). Similarly, no significant effect of supplementation condition or of training session were observed for the [Hcy] during the weightlifting training session (*p* > 0.05).

### Effect of POMj on the delayed response of plasma Hcy and steroidal hormones at 48 h following the weightlifting training session

Table 3 shows the values of the steroidal hormones, the [T/C] ratio and the [Hcy] before, immediately (3 min), and 48 h after the training sessions during the PLA and POMj trials. From 3 min to 48 h after the training-session, plasma [Hcy] decreased significantly only in the POMj condition (*p* = 0.04 and Δ rate of decrease = − 14 ± 12.9%). However, both PLA and POMj condition showed a significant increase in plasma [C] (i.e., *p* = 0.002, Δ rate of increase = 99.27 ± 69.3% and *p* = 0.02, Δ rate of increase = 78.09 ± 89.0%, respectively) and a significant decrease in [T/C] ratio (i.e., *p* = 0.001, Δ rate of decrease = − 47.5 ± 15.2% and *p* = 0.02, Δ rate of decrease = − 26.1 ± 39.7, respectively).

**Table 3:** Recovery kinetics of Homocysteine, the steroidal hormones (i.e.*,* cortisol (C) and testosterone (T)) and T/C ratio responses after placebo (PLA) and pomegranate (POMj) supplementation

Variables	PLA				POMj			
	Pre-session	3 min post-session	48 h of recovery	Δ 48 h-3′ (%)	Pre-session	3 min post-session	48 h of recovery	Δ 48 h-3′ (%)
***Homocysteine (μmol/L)***	12.57 ± 3.55	12.72 ± 2.87	12.85 ± 2.73	*P* = 0.75	12.85 ± 2.73	13.99 ± 2.51	12.42 ± 2.64 **+**	−14.12 ± 4.31
***Testosterone (ng/mL)***	5.84 ± 1.00	5.92 ± 0.58	5.94 ± 0.92	*P* = 0.84	5.94 ± 0.92	5.41 ± 0.61	5.63 ± 0.66	*P* = 0.46
***Cortisol (ng/mL)***	110.11 ± 12.03	58.22 ± 7.50	108.56 ± 9.89 **+**	99.27 ± 23.11	108.56 ± 9.89	65.67 ± 9.49	98.78 ± 9.83 **+**	78.09 ± 29.67
***T/C ratio***	0.06 ± 0.01	0.11 ± 0.01	0.06 ± 0.01 **+**	−47.54 ± 5.06	0.06 ± 0.01	0.09 ± 0.01	0.06 ± 0.01 **+**	− 26.11 ± 13.2

**+**: Significant differences between 48 h and 3 min post training session

For all tested parameters, no significant difference was shown between PLA and POMj in plasma levels 48 h post training session (*p* > 0.05). Similarly, for all tested parameters, no significant differences were observed between the plasma levels registered at pre- and 48 h post- exercise sessions indicating that in both conditions, 48 h recovery period was sufficient to recover the baseline values of [Hcy], [T] and [C] (*p* > 0.05).

### Relationships between Hcy and the steroidal hormones measures

Table 4 shows the relationship between the measures (pre- and post- training session during PLA and POMj conditions) of [Hcy] and the steroidal hormones. The Hcy measures showed only a significant moderate negative correlation with [T] (*r* = − 0.50 and *p* = 0.002). Concerning the inter-relation between the steroidal hormones measures, a significant low correlation was found between [T/C] ratio and [T] (*r* = 0.04 and *p* = 0.02), while a significant moderate negative correlation was found between [T/C] ratio and [C] (*r* = − 0.05 and *p* = 0.003) with no significant correlation was found between [T] and [C] (*p* > 0.05).

**Table 4:** Relationship between Homocysteine, the steroidal hormones (i.e.*,* cortisol (C) and testosterone (T)) and T/C ratio measures

Variables	Correlation
***Relationship between Hcy and the steroidal hormones***	
Hcy-Testosterone	*r* = −0.5, *p* = 0.002
Hcy-Cortisol	*p* = 0.13
Hcy-T/C rati	*p* = 0.16
***Inter-relationship between the steroidal hormones***	
Testosterone-Cortisol	*p* = 0.10
T/C ratio-testosterone	*r* = 0.4, *p* = 0.02
T/C ratio-Cortisol	*r* = −0.5, *p* = 0.003

## Discussion

The aim of the present study was to investigate the effect of a specific supplementation protocol (i.e., repeated doses of 250 ml during the 48 h proceeding the training session combined with an acute dose of 500 ml 1 h before the training session) with a natural POMj on the acute and delayed [Hcy] and steroidal hormone responses following a weightlifting training session. In response to the training session, circulating [C] decreased and [T/C] increased in both PLA and POMj conditions, while [T] decreased only following POMj supplementation (− 9.8%). During the 48 h recovery period, all tested parameters were shown to recover to baseline values in both conditions with significant increases in [C] and decreases in [T/C] from 3 min to 48 h post-exercises. The slight increase in [Hcy] immediately following exercise, returned to baseline during the 48 h recovery period in the POMj condition, with the acute and delayed responses of [Hcy] shown to be negatively correlated (*r* = − 0.5) to [T] responses.

In sport, testosterone is a recognized androgen which maintains anabolism by promoting bone development and protein synthesis within the musculoskeletal system [61]. By contrast, cortisol is a glucocorticoid (responsible for 95% of glucocorticoid activity) released under physiological and/or psychological stress [62, 63]. Most often, cortisol is viewed as having a counter-productive role in exercise that can lead to a maladaptation to the exercise training process due to the catabolic effect of this hormone on skeletal muscle [64]. Therefore, decreases in circulating [T] (i.e.*,* decrease) and/or increases in [C] can lead to an imbalance between anabolism and catabolism in favor of the latter [65] which, in turn, can negatively affect strength and muscle adaptations associated with resistance exercise performance [66].

In order to monitor anabolic/catabolic balance during strength exercise, previous studies have investigated circulating [T] and [C] before and after strength exercises, but the existing findings are inconsistent. Indeed, while [T] increased significantly from pre to post training session in the studies of Weiss et al. [67] (ΔT: 22%), Jensen et al. [68] (ΔT: 27%), Crewther et al. [44] (ΔT: 7%) and Ammar et al. [42] (ΔT: 12%), [T] remained unchanged in other reports [50, 69, 70]. Surprisingly, Hakkinen et al. [71] reported that, following successive strength sessions performed in 1 day, [T] may increase, decrease or not change.

Similarly, divergent findings have been reported for ΔC during strength exercises. Indeed, in response to a high-volume lower body resistance exercises session [50], or a high-intensity weightlifting training session [44], [C] was showed to increase by 11% from pre to post exercise-session [44]. However, following a hypertrophy based-exercises-protocol, [C] decreased significantly (ΔC: − 24.7%) compared to the baseline values [42] which can result in higher post exercises values for [T/C] ratio (+ 60.3%) [42].

By showing unchanged [T] among elite athletes across a weightlifting session in the PLA condition, the present findings support the results of Guezennec et al. [69] and Crewther et al. [70] suggesting that strength exercise does not appear to provide sufficient stimulus to induce significant increases in [T]. However, the present decrease in [C] and increase in [T/C] ratio in both PLA and POMj conditions confirms that strength exercise is one of the best training modalities to evoke hormonal changes with the potential to enhance muscle strength and adaptation. In fact, and in contrast to the present result, a rise in [C] has been reported to stimulate skeletal protein degradation [72] and inhibit the neuromuscular system [73], which may promote a catabolic environment. Increased [T/C] ratio, on the other hand, has previously been suggested to reduce and promote anabolism [42] with beneficial implications for strength development and training adaptation [72].

The discrepancies between these findings could be due to factors related to the training session such as relative intensity to the maximum load (% of 1 repetition maximum [1RM]) [74], number of sets [75] and solicited muscle mass [42]; with a catabolic state likely occur when a large volume of training is performed [76], while an anabolic state may occur when a large mass of skeletal muscle is engaged [42]. Additionally, factors related to the population were investigated, such as training status [65], which has also been demonstrated to exhibit large individual variability in ΔC (e.g., − 57 to 255%) and ΔT (e.g.*,* − 67 to 126%) [44, 70]. It appears that the pleiotropic effects of T on the neuromuscular system, directly lowers the magnitude of the acute adrenal cortical response to strength exercise [70] compared to untrained subjects [77]. In accordance with this suggestion, the present findings using elite weightlifters confirm training status as an influencing factor and showed that elite athletes were unable increase [T] in response to the PLA weightlifting exercises.

Regarding the effect of POM supplementation on the tested parameters, the present results demonstrated that [T] decreased significantly from pre- to post- exercise only during the POMj condition with no significant difference between PLA and POMj in the [C] and [T/C] responses. These findings are not consistent with those of Al-Dujaili and Smail [19]. who reported a significant increase in blood [T] following 2 weeks of POMj intake in 60 healthy males and females. However, the present findings are consistent with previous studies indicating that POMj consumption inhibits Tproduction in 38 normal weight postmenopausal healthy women [78], in 23 patients with Polycystic Ovarian Syndrome [79] and among rats with Benign prostatic hyperplasia [80]. Similarly, our results are in line with recent study in healthy untrained men showing that, compared to PLA, polyphenol supplementation slightly attenuate acute plasma [T] response with no observed effect on [C] response following strength exercise [50].

The exact mechanisms explaining the post-exercise decrease in plasma [T] following POMj intake still not well established [78]. However, two main phenomena are suggested to explain the present impairment of plasma [T] response using POMj. On the one hand, the decrease in post-exercise plasma [T] using POMj may be due to a decrease in total T production. Indeed, POM components, specially flavonoids, hydrolyzable tannins and catalpic and punicic acids (a unique conjugated a-linolenic acid) have been purported to inhibit T production by decreasing gene expression and androgen receptor content [81] most consistently in the LNCaP-AR cell line [15] and by decreasing cytochrome P450C17 [82] possibly through the improvement of insulin resistance, insulin levels and fasting glucose concentrations [79] by upregulating the expression of peroxisome proliferator-activated receptors (PPARs) in several different tissues such as the abdominal adipose tissue [83]. Based on this explanation, and taking in consideration that decreasing T production favors a more catabolic environment [65, 66], the present decrease in plasma T levels and probably in total T production using POMj supports previous reports indicating that strong antioxidants taken close to the exercise stimulus might actually impair anabolic-catabolic balance and thereby attenuate training adaptations in response to both endurance [84, 85] and strength training [86]. In this context, it was previously shown that antioxidant supplements such as quercetin, vitamin C and/or vitamin E attenuate the increase in mitochondrial biogenesis after endurance training [84, 85] and significantly blunt strength gains after 10 weeks of resistance training in recreationally active men [86]. On the other hand, the present reduction in the plasma post-exercise T using POMj could be caused by increased uptake of T (primary androgen receptor (AR)-activating hormone [87]) into skeletal muscle during exercise to bind to the androgen receptor and thereby provide an increased anabolic stimulus to promote bone development and protein synthesis within the muscular system [61]. If confirmed, polyphenol-rich nutrients might have the potential to improve acute and chronic adaptations to resistance exercises without limiting muscular training gains. In this context, previous research has reported that 4 weeks consumption of antioxidant nutrients coupled with eccentric training (i.e., using vitamin C and E [88]) or muscular endurance resistance training (i.e., using green tea extract [89]) did not affect gains in isometric strength [88] and did not alter the changes in one repetition maximum [89]. More recently, Beyer et al. [50] have provided additional evidence that antioxidant nutrients are less likely to attenuate training adaptation and showed that, flowing a 6-week progressive resistance training program using PLA or polyphenol blend (PPB) supplementation, no significant difference in maximal strength gains was observed between conditions during squat, leg press, and leg extension exercise. The conflicting findings of previous research coupled with the different explanation and interpretation of decreasing post exercise plasma [T] using POMj compared to PLA render the usefulness of antioxidant supplementation during strength training as questionable at this stage. To resolve the functional consequences of this effect, further longitudinal studies investigating the effect of chronic rich-polyphenol administration during strength training on both plasma and muscular [T] are warranted.

The delayed response of the tested parameters is in agreement with studies conducted by Häkkinen et al. [71], Jensen et al. [68] and Beyer et al. [50] who found that serum concentrations of steroidal hormones declined back to pre-training baseline levels after just 2 h to 1 day of rest. The present findings showed in both the PLA and POMj conditions a significant increase in [C] and a significant decrease in [T/C] ratio during the 48 h recovery period which allowed these parameters to reach their baseline levels. Similarly, [Hcy] was shown to recover to baseline values 48 h post exercise in both the PLA and POMj conditions following a decrease during the 48 h recovery period following POMj supplementation. The reduction in [Hcy] from 3 min to 48 h post-exercise was only observed in the POMj condition and does not support previous findings showing a potential beneficial effect of polyphenol-rich beverages in attenuating [Hcy] in healthy individuals and patients with Alzheimer’s disease [45]. Indeed, this reduction in [Hcy] occurred during the 48 h recovery period to amend the slight increase of Hcy observed immediately following exercise using POMj. The recovery of baseline values for all tested parameters 48 h post exercise in both the PLA and POMj conditions confirms this interpretation and suggest no potential effect of POMj on the delayed responses of the tested parameters.

The present results also showed a significant negative relationship between [Hcy] and circulating [T]. This association is in line with previous findings suggesting [T] as independent predictor and regulator of plasma [Hcy] concentration in both health and diseases individuals [90, 91]. Hcy has recently gained attention in research as CVD risk factor that can independently predict the risk of this disease [92]. Indeed, with each additional 5 μmol/L increase in [Hcy], the risk of CVD events is increased by approximately 20% [93]. Accordingly, maintaining low [Hcy] can protect cerebral vessels and can prevent the accumulation of DNA damage caused by oxidative stress and facilitated by [Hcy] [45]. Therefore, the present association supports recent research indicating that androgens may be novel CVD risk factors and potential predictors of human health problems [94]. However, to confirm these postulates, further studies with a higher number of elite weightlifters and investigating large androgens and direct CVD risk-factor parameters are needed.

Taken together, these preliminary findings suggest a possible beneficial effect of dietary intake of natural polyphenol-rich POMj (250 ml POMj × 3 times daily) on acute plasma [T] response following high-intensity strength exercise in an athletic population. However, given that the potential applications of this work (e.g., dose and form of POMj, physiological responses etc.) are most relevant for an athletic population, further large-scale research in both athletic and non-athletic populations is needed to corroborate these preliminary observations and to elucidate the potential underlying mechanisms and translational potential of our novel observations in the current study.

## Conclusions

This study is the first to test the efficacy of POMj supplementation on [Hcy] and circulating steroidal hormones. Supplementation with POMj has the potential to attenuate the acute plasma [T] response, suggested to predict and regulate plasma [Hcy], but with no potential effect on the recovery kinetics of the tested parameters as all parameters returned to baseline values 48 h post exercise in both the PLA and POMj conditions. Additional large, randomized trials considering a longer intervention period with different doses of POM intake and investigating large androgens and inflammatory cytokines in both plasma and muscle tissue are warranted to resolve the functional consequences of the observed acute POMj effect. Since resistance training is now recommended to enhance health outcomes in the general population [95], further research should also investigate non-athletic populations.

## Data Availability

The data supporting the present findings can be requested from the correspondent author via e-mail (ammar.achraf@ymail.com).

## Abbreviations

1-RM: 1-Repetition Maximum
AR: Androgen receptor
C: Cortisol
CVD: Cardiovascular disease
ES: Effect size
GA: Gallic acid
HB: Hemoglobin
HCT: Hematocrit
Hcy: Homocysteine
PLA: Placebo
POM: Pomegranate
POMj: Pomegranate juice
PPARs: Peroxisome proliferator-activated receptors
RBC: Red Blood Cells
T: Testosterone
T/C: Testosterone/Cortisol ratio
η^2^: Eta squared
η_p_^2^: Partial eta-squared
